# Pathogenic variant c.1052T>A (p.Leu351Gln) in adenosine deaminase 2 impairs secretion and elevates type I IFN responsive gene expression

**DOI:** 10.3389/fimmu.2022.995191

**Published:** 2022-09-30

**Authors:** Sarah M. Bowers, Martina Sundqvist, Paul Dancey, David A. Cabral, Kelly L. Brown

**Affiliations:** ^1^ Department of Microbiology and Immunology, University of British Columbia, Vancouver, BC, Canada; ^2^ British Columbia Children’s Hospital Research Institute, Vancouver, BC, Canada; ^3^ Janeway Children’s Health and Rehabilitation Centre, Saint John’s, NL, Canada; ^4^ Department of Pediatrics, University of British Columbia, Vancouver, BC, Canada; ^5^ British Columbia Children’s Hospital, Vancouver, BC, Canada

**Keywords:** adenosine deaminase 2 (ADA2), type I Interferon (IFN), systemic vasculitis, pediatric, inflammation

## Abstract

**Background:**

Adenosine deaminase 2 (ADA2) is a homodimeric, extracellular enzyme and putative growth factor that is produced by cells of the myeloid lineage and, catalytically, deaminates extracellular adenosine to inosine. Loss-of-(catalytic)-function variants in the *ADA2* gene are associated with Deficiency of ADA2 (DADA2), an autosomal recessive disease associated with an unusually broad range of inflammatory manifestations including vasculitis, hematological defects and cytopenia. Previous work by our group led to the identification of *ADA2* variants of novel association with DADA2, among which was a unique c.1052T>A (p.Leu351Gln; herein referred to as L351Q) variant located in the catalytic domain of the protein.

**Methods:**

Mammalian (Flp-IN CHO) cells were engineered to stably express wild-type ADA2 and ADA2 protein variants, including the pathogenic L351Q variant identified in DADA2 patients. An enzyme assay and immunoblotting were used to assess ADA2 catalytic activity and secretion, respectively, and the outcome of experimentally induced inhibition of protein processing (Golgi transport and N-linked glycosylation) was assessed. Reverse transcription quantitative real-time PCR (RT-qPCR) was applied to determine the relative expression of Type I Interferon stimulated genes (ISGs), *IFIT3* and *IRF7*.

**Results:**

In addition to abrogating catalytic activity, the L351Q variant impaired secretion of L351Q ADA2 resulting in an intracellular accumulation of L351Q ADA2 protein that was not observed in cells expressing wild-type ADA2 or other ADA2 protein variants. Retention of L351Q ADA2 was not attributable to impaired glycosylation on neighboring asparagine residues and did not impact cell growth or integrity. Constitutive expression of Type I ISGs *IFIT3* and *IRF7* was observed in cells expressing L351Q ADA2.

**Conclusions:**

The impaired secretion of L351Q ADA2 may be an important factor leading to the severe phenotype observed in patients with this variant further emphasizing the importance of assessing impacts beyond catalytic activity when evaluating genotype-phenotype relationships in DADA2.

## Introduction

Adenosine deaminase 2 (ADA2) is a homodimeric, extracellular enzyme and putative growth factor that is produced by cells of the myeloid lineage and, catalytically, deaminates extracellular adenosine to inosine. In the absence of a rodent ortholog, most knowledge about ADA2 has come since 2014 when the first cases of a clinical syndrome were described in two independent cohorts of children who were found to have ‘Deficiency of ADA2’ (DADA2). These patients were previously diagnosed with a medium-sized vessel vasculitis called polyarteritis nodosa (PAN) (1, 2). DADA2 is an autosomal recessive disease caused by loss-of-(catalytic)-function variants in the *ADA2* gene. Although vasculitis (i.e., inflammation of blood vessels) is a predominant feature, early-age of onset is characteristic and, in the majority of DADA2 patients, the disease also has an unusually broad range of inflammatory manifestations including hematological defects (e.g., cytopenia), and immunodeficiency (1–5).

The *ADA2* gene is highly polymorphic and since the initial reports of DADA2, over 100 disease-causing variants associated with DADA2 have been described (4, 6). All DADA2-associated variants have a damaging effect on catalytic activity, yet not all variants are restricted to the catalytic domain. In fact, pathogenic variants have been identified across the entire coding region (7, 8) and in each of the predicted domains of the protein (4, 9) inclusive of the catalytic domain as well as the signal sequence, dimerization, and putative receptor binding domains. Genotype-phenotype correlations in DADA2 have been difficult to establish given the genotypic and phenotypic diversity, and this difficulty is compounded by an incomplete understanding of the physiologic function of human ADA2.

Studies that have explored an association between particular variants and clinical features have tended to focus on the specific location of variants and/or the precise protein domain within which the variants reside (5, 6, 10, 11). More recently, work by Lee and colleagues evaluated the nature of the mutation and suggested that, regardless of the location in the *ADA2* gene, missense mutations that allow residual catalytic activity are more frequently associated with vasculopathy, whereas bone marrow failure and pure red cell aplasia in DADA2 patients are more frequently associated with nonsense variants, insertions/deletions and particular missense variants in *ADA2* leading to abrogation (<3% of normal) of catalytic activity (12). The functional impacts of ADA2 dimerization, post-translational modifications (namely N-linked glycosylation) and secretion have not been explored for all variants and their unique roles in the development of distinct disease features have not been considered.

Previous work by our group led to the identification of ADA2 variants of novel association with DADA2, among which was a unique c.1052T>A (p.Leu351Gln; herein referred to as L351Q) variant located in exon 7 of the *ADA2* gene and within the predicted catalytic domain. Patients with this missense variant suffered from severe, early-onset neurologic, inflammatory, and hematological manifestations in the absence of vasculopathy. Other DADA2 patients with variants resulting in an amino acid substitution in close proximity to the L351Q variant, including a patient compound heterozygous for Y353H (13), and homozygous for the G321E variant (14), also presented with neutropenia. Together, these observations may indicate a role for variants in this region of the ADA2 protein in contributing to hematologic defects of DADA2 independent of vascular inflammation.

To assess the impact of the L351Q variant of novel association with DADA2 on ADA2 protein structure and function, we generated CHO cell lines using the Invitrogen™ Flp-In System™ that stably express recombinant human wild-type (WT) and L351Q ADA2. By way of comparison, we also generated CHO cell lines expressing ADA2 containing a dimerization domain-localized variant, G47R, which is associated most frequently with vasculopathy in DADA2 (6). Consistent with previous reports (1, 2), the expression and secretion of G47R ADA2 was compromised (compared to WT ADA2) and especially so when N-linked glycosylation was inhibited. Our findings also demonstrate that (in addition to being catalytically inactive) L351Q ADA2, but not WT or G47R ADA2, forms dimers and is highly stable with or without incorporation of N-linked glycans. Additionally, and unlike WT and G47R ADA2 that are readily secreted from the CHO cells, our data reveals a striking impairment in the secretion of L351Q ADA2, resulting in an intracellular accumulation of L351Q ADA2 protein and constitutive expression of Type I Interferon stimulated genes (ISGs). These functional consequences of the L351Q variant (impaired secretion and elevated ISG expression) may, in addition to impairment of ADA2 catalytic activity, contribute to the severe phenotype observed in patients with this variant.

## Materials and methods

### *In vitro* expression of ADA2 protein variants

Reagents are products of Invitrogen (Thermo Fisher Scientific, MA, USA) unless otherwise specified. In brief, the Q5^®^ site directed mutagenesis (SDM) kit (NEB, MA, USA) was used to introduce the c.139G>C (p. Gly47Arg [G47R]), c.1052T>A (p.Leu351Gln [L351Q]), and c.1084T>G (p.Trp362Gly [W362G]) variants to wild-type (WT) human *ADA2* (ENST00000399839.1, Ensembl GRCh37) with a C-terminal 6xHis tag sequence inserted in a pcDNA5/Flp Recombination Target (FRT) vector construct by GenScript (NJ, USA). Non-overlapping mutagenic primers (GenScript, NJ, USA) were used for SDM (**Supplementary Table S1**). The variant construct sequences were verified by Sanger sequencing targeting the multiple cloning site (CMMT/BCCHR DNA Sequencing Core Facility, Vancouver, BC) and plasmids for transfection were isolated using the GeneJET Plasmid Midiprep Kit (Thermo Scientific, MA, USA).

Chinese hamster ovary (CHO) cells stably expressing WT and the G47R, L351Q, and W362G variant forms of ADA2 protein were generated using the Flp-IN™ system (Invitrogen by Thermo Fisher Scientific, MA, USA) as directed by manufacturer’s instructions. In brief, Flp-IN CHO cells (R75807) containing a single stably integrated FRT site were transfected at confluency with the pOG44 plasmid encoding Flp-Recombinase and the pcDNA5/FRT expression vector constructs (encoding either WT, G47R, L351Q, or W362G ADA2) using lipofectamine 2000. Transfected Flp-IN CHO cell lines were maintained in tissue culture treated flasks in Ham’s F12K media supplemented with 10% heat inactivated fetal bovine serum (FBS), 2 mM L-glutamine, and 400 µg/mL Hygromycin B selection reagent and passaged at 80-90% confluency. For maintenance of untransfected Flp-IN CHO cells, media was supplemented with 100 µg/mL Zeocin™ selection reagent (no Hygromycin B) (all products of Gibco by Thermo Fisher Scientific, MA, USA). Where indicated, cells were incubated in the presence of Tunicamycin (0.1-10 µg/mL of media; Cell Signaling Technology, MA, USA) or GolgiStop™ Protein Transport Inhibitor containing monensin (0.35-5.6 µL GolgiStop™/mL of media; BD Biosciences, MA, USA) for 6 hr prior to ADA activity assay and immunoblotting.

### Adenosine deaminase activity assay

An ADA activity assay (Diazyme Laboratories Inc., CA, USA) modified as described previously (3, 15) was used to measure ADA enzyme activity in Flp-IN CHO cell supernatant/lysate following 6 hr of culture at 2x10^6^ cells/mL in fresh media (0.25 mL). Following culture, supernatant was removed from adherent cells and prepared by addition of Halt™ protease inhibitor cocktail (Thermo Scientific, MA, USA) and centrifugation at 300xg; cleared supernatant was used immediately or stored at -80°C until use. Remaining adherent cells were washed then lysed directly in the culture dish, or trypsinized then lysed, on ice for 10 min at approximately 2x10^6^ cells/mL with 0.25 mL of non-denaturing buffer (PBS with 1% Triton X-100 and protease inhibitor cocktail). Lysate was cleared by centrifugation (16,000xg for 10 - 25 min) at 4°C and was used immediately or stored at -80°C until use. Extracellular and intracellular ADA activity was captured by analysis of 10 uL of cell culture supernatant (extracellular ADA activity from 2x10^4^ cell equivalents) and cell lysate (intracellular ADA activity in 2x10^4^ cell equivalents). To measure ADA2 specific activity, erythro-9-(2-hydroxy-3-nonyl)adenine hydrochloride (EHNA; Millipore Sigma, MA, USA), an ADA1 specific inhibitor was added (17.54 μM final concentration in the assay). One unit of ADA activity is defined as the amount of enzyme required to produce one μmole of inosine per minute under assay conditions.

### Immunoblotting

Immunoblotting was used to identify ADA2 protein in Flp-IN CHO cells/supernatant following 6 hr of culture at approximately 6x10^6^ cells/mL in fresh media (0.4 mL; no FBS, no antibiotics). Supernatant and lysates were prepared and stored as described for the ADA2 enzyme activity assay except cells were lysed at approximately 1.6x10^7^ cells/mL with 0.15 mL of RIPA buffer containing 1% NP-40 (Cell Signaling Technology, MA, USA) and protease inhibitor cocktail. SDS-PAGE (4-12% NuPAGE™ Bis-Tris gels; Invitrogen, MA, USA) was used to resolve 18.75 µL of: cell culture supernatant (extracellular ADA protein from 1.125x10^5^ cell equivalents) and cell lysate (intracellular ADA protein in 3x10^5^ cell equivalents). Recombinant human (rh) His-tagged ADA2 (50 ng; R&D systems, MN, USA) was included as a control where indicated. For immunoblotting, samples were transferred to a polyvinylidene difluoride (PVDF) membrane (Millipore Sigma, MA, USA) and membranes were blocked in tris-buffered saline with 0.05% Tween 20 and 5% bovine serum albumin. Antibodies are products of Invitrogen (Thermo Fisher Scientific, MA, USA) unless otherwise specified. Membranes were probed with rabbit anti-ADA2 polyclonal antibody (1:1,000; PA5-30635), mouse anti-6xHis monoclonal antibody (1:1,000; MA1-21315), rabbit anti-GPR78 polyclonal antibody (1:2,000; PA1-014), or rabbit anti-β-actin monoclonal antibody (1:1,000 - 2,000; 8457; Cell Signaling Technologies, MA, USA) followed by IRDye 800CW goat-anti-rabbit IgG and/or 680CW "goat-anti-mouse lg secondary" antibody (1:20,000; LI-COR Biosciences, NE, USA) and imaged using the LI-COR Odyssey imaging system. Protein bands were compared to the PageRuler™ prestained protein ladder (Thermo Fisher Scientific, MA, USA) for relative molecular weight and images were processed using ImageJ (v. 1.52k; NIH, USA). Relative abundance of ADA2 protein was calculated from densitometry analysis of ADA2 monomers and dimers, with signal normalized to 50 ng rhADA2 as a control. Relative abundance of ADA2 protein in the supernatant was corrected for the differential abundance of proteins resolved by SDS-PAGE from supernatant versus lysate (approximately 1:2.67 [SN:lysate] based on cell equivalents) and total ADA2 protein was calculated indirectly (SN + lysate).

### Flp-IN CHO cell stimulation and RT-qPCR

Flp-IN CHO cells were seeded in tissue culture treated plates (2x10^5^ cells per well in 12-well plates) and grown overnight (16 hr). Recombinant human (rh) Type I IFNalpha 2b protein (Novus Biologicals, CO, USA) was added to the culture (10 ng/mL media, no antibiotics) for 24 hr. The RNeasy mini kit (Qiagen, DE, USA) was used according to manufacturer’s instructions to lyse Flp-IN CHO cells and isolate RNA, with the RNase-free DNase Set (40.9 Kunitz units/sample; Qiagen, DE, USA) for on column DNA digestion (25 min). RNaseOUT Recombinant Ribonuclease Inhibitor (40 units/sample; Invitrogen by Thermo Fisher Scientific, MA, USA) was added to RNA preparations. A NanoDrop ND-1000 spectrophotometer (Thermo Fisher Scientific, MA, USA) was used to measure RNA concentration and purity. The qScript cDNA Synthesis Kit (QuantaBio, MA, USA) was used for synthesis of cDNA (500 ng RNA input). TaqMan Fast Advanced Mastermix and gene expression assays (Applied Biosystems, MA, USA) were used to measure expression of CHO *IFIT3* (Assay: Cg04640028_s1), *IRF7* (Assay: Cg04575257_g1) and eukaryotic *HPRT1* (Assay: Hs03929096_g1). Assays were run in duplicate on fast optical 96-well plates (Applied Biosystems, MA, USA) using a QuantStudio 6 Real-Time PCR instrument (Thermo Fisher Scientific, MA, USA). Reactions contained 2 μl of cDNA in a total reaction volume of 10 μl, and thermal cycling conditions were 2 min at 50°C, 2 min at 95°C, 40 cycles of 1 s at 95°C and 20 s at 60°C. For *IFIT3* and *IRF7*, relative gene expression was calculated using the formula 2^−ΔCt^ with the mean Ct of *HPRT1* as the housekeeping control.

### Statistical analysis

Statistical analyses were done using GraphPad Prism statistical software (v9.0; GraphPad Software, CA, USA). Group differences were analyzed by ANOVA and subsequent parametric multiple comparisons tests, as indicated in figure legends. For all analyses, a confidence interval of 95% was used; a p-value < 0.05 was considered significant.

## Results

### Variant c.1052T>A (p.Leu351Gln) in the *ADA2* gene impairs L351Q ADA2 protein secretion

**Figure 1:**
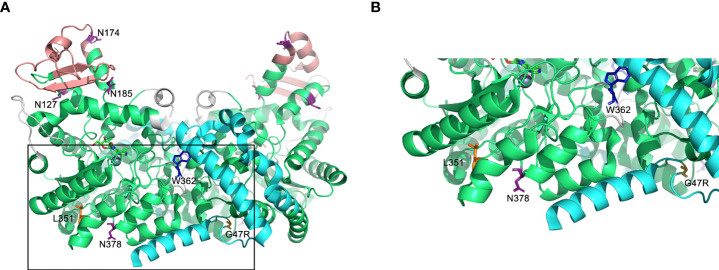
Predicted protein structure of human ADA2. **(A)** A ribbon diagram of the ADA2 homodimer crystallized in the presence of coformycin (transition state analog) and zinc (cofactor) using data available from the Protein Data Bank (PDB 3LGG) (16). The catalytic domain, dimerization domain, putative receptor binding (PRB) domain, and small unique elements are shown in green, cyan, light red, and grey, respectively. Predicted sites of N-linked glycosylation are labeled (N127, N174, N185, and N378) on one monomer, and the relevant asparagine residues are shown in purple (16, 17). Leucine 351 (L351) and tryptophan 362 [W362; W336 in (16)] are labelled and shown in orange and blue, respectively, on one monomer and glycine 47 (G47) is labelled and shown in yellow on the other monomer. The boxed region of A is expanded in **(B)**. Figures were generated using PyMOL version 2.4.0 (Schrödinger, Inc., NY, USA).

We generated (Flp-IN) CHO cell lines that stably expressed recombinant human wild-type (WT) ADA2 and two pathogenic loss-of-(catalytic) function variants located in different domains of the protein (**Figure 1**) and associated with different clinical phenotypes: G47R is a variant frequently associated with DADA2 with vasculopathy being a common feature (12), and L351Q is a variant we discovered in two DADA2 patients (siblings) that, unlike the G47R variant, was associated with severe, early-onset disease that was fatal for one child. Predominant features were neutropenia, cerebral atrophy and stroke in the absence of vasculopathy (3). By way of comparison, we also generated Flp-IN CHO cells expressing ADA2 with the point mutation W362G. This variant has not been reported, to date, in DADA2 patients, but has demonstrated a greater disassociation rate *in vitro* to monomeric form (16). Of note, the W362G variant is numbered W336G in the crystal structure presented by Zavialov et al., which lacks the signal sequence (16). Despite being noncontiguous with the dimerization domain subsequence, wild-type W362 mediates the ‘tryptophan-catch’ intersubunit contact between ADA2 monomers [W336 in (16)]. Thus, we refer to W362G as a variant in the dimerization domain which is defined by its functional role in connecting subunits of the dimer (16).

**Figure 2:**
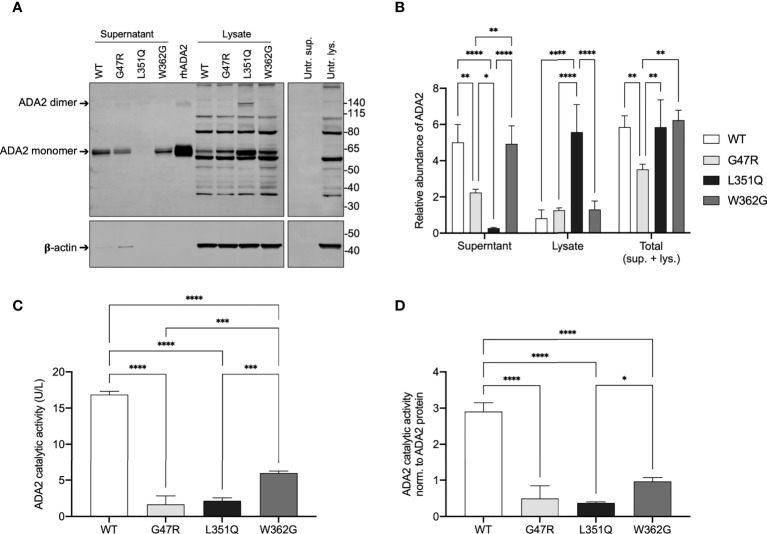
ADA2 protein expression and catalytic activity in CHO cells transfected with expression vectors for wild-type and variant ADA2. **(A)** Representative anti-6xHis immunoblot (upper panel) and beta-actin (β-actin; loading control for lysates, lower panel) of supernatant and lysate (3x10^5^ cell equivalents) from Flp-IN CHO cells untransfected (Untr.) and transfected with expression vectors for His-tagged wild-type (WT) and variant (G47R, L351Q, W362G) adenosine deaminase 2 (ADA2) (n = 3). Recombinant human (rh) 6xHis-tagged rhADA2 (50 ng rhADA2) is included for comparison as is untransfected cell supernatant (Untr. sup.) and lysate (Untr. lys.). Ladder (left) marks relative molecular weight in kilodaltons (kDa). Arrows show ADA2 monomers (~60 kDa) and dimers (>120 kDa). **(B)** Graph of relative protein abundance (y-axis; Relative abundance of ADA2, n = 3) based on densitometry analysis of WT and variant ADA2 in cell supernatant (x-axis; Supernatant), lysate (x-axis; Lysate), and the calculated total from supernatant and lysate combined (x-axis; Total (sup. + lys.)), visualized by anti-6xHis and anti-β-actin immunoblots (as shown in A), with signal normalized to 50 ng of rhADA2 included on each gel (see Methods). **(C)** Total ADA2 catalytic activity (y-axis; ADA2 catalytic activity (U/L), n = 3) in supernatant and lysate from Flp-IN CHO cells transfected with expression vectors for WT (x-axis; WT, white) and variant (x-axis; G47R, light grey; L351Q, black; W362G, dark grey) *ADA2*. **(D)** Total ADA2 catalytic activity shown in **(C)**, normalized to the total relative abundance of ADA2 protein (sup. + lys.) shown in **(B)** (y-axis; ADA2 catalytic activity norm. to ADA2 protein, n = 3). Bars show mean + SD and statistics show significant results of Tukey’s multiple comparisons test to compare group means. *p ≤ 0.05, **p ≤ 0.01, ***p ≤ 0.001, ****p≤ 0.0001.

Protein production and secretion of His-tagged WT and variant ADA2 by stably transfected CHO cell lines were initially evaluated by anti-6xHis immunoblot of the cell supernatants (extracellular ADA2 protein from approximately 1.125x10^5^ cell equivalents) and lysates (intracellular ADA2 protein in approximately 3x10^5^ cell equivalents). Commercially available, CHO cell-derived recombinant human (rh) ADA2 with a 6xHis tag was included as a control and afforded a strong signal (band) at approximately 60 kDa and a substantially less intense, 115 – 140 kDa band, which is consistent with the predicted molecular weight, respectively, of an ADA2 monomer (58.9 kDa; Uniprot Identifier Q9NZK5-1) and homodimer (approximately 120 kDa). Untransfected CHO cell supernatant and lysate were also included as controls and revealed (by anti-6xHis immunoblot) multiple protein bands in the lysate, but not supernatant (**Figure 2A**). These bands are most likely endogenous, histidine repeat-containing CHO cell proteins as they were not present in CHO cell lysates subjected to immunoblotting with anti-ADA2 antibodies. Anti-ADA2 immunoblotting of untransfected CHO cell lysate was also negative for proteins at molecular weights corresponding to ADA2 monomers and homodimers (**Supplementary Figure S1A**). A putative 48 kDa protein was, however, detected in untransfected CHO cell lysates probed with anti-ADA2 antibodies (**Supplementary Figure S1A**). While the identity of this intracellular protein is unknown, it does not appear to have ADA enzyme activity as negligible ADA (total) and ADA2-specific enzyme activity was measured in untransfected CHO cells (**Supplementary Figure S1B**). This is consistent with the absence of an *ADA2* ortholog in rodents (18), and an advantage of using CHO cells as a mammalian expression system for ADA2 compared to other cell lines (e.g. U937) that express endogenous ADA2 [proteinatlas.org (19)].

As expected for a secreted protein, ADA2 was present predominantly in the supernatant of WT "ADA2 expressing" CHO cells, with extracellular/secreted WT ADA2 protein (in supernatant) representing 85% of the total (in supernatant + lysate) WT ADA2 protein (n = 3) (**Figure 2A** and quantitated in **Figure 2B**). CHO cells transfected with dimerization domain-localized variant W362G ADA2 produced a similar total (supernatant + lysate) quantity and similar distribution (supernatant/lysate) of ADA2 protein as WT ADA2 expressing cells. In contrast, the total quantity of dimerization domain-localized variant G47R ADA2 protein was significantly reduced compared to WT (61% of total WT ADA2 protein, n = 3, *p* = 0.0081) and predominantly so in the supernatant (47% of WT ADA2 protein in supernatant, n = 3, *p* = 0.0016). These data are consistent with speculations that dimerization is required for ADA2 secretion and/or stability. However, it was not evident from our analyses if ADA2 containing variants W362G or G47R display the expected propensity to form monomers given that WT ADA2 (commercial and in-house), used for comparison, was itself predominantly monomeric. An almost identical finding of reduced G47R ADA2 secretion by HEK293 cells was reported by Navon-Elkan et al. (1) and is consistent with the observation by Zhou et al. of substantially reduced ADA2 intracellular and extracellular protein in peripheral blood derived macrophages from a patient with heterozygous G47A/H112Q ADA2 compared to the carrier mother (WT/H112Q) (2).

The most striking result from the immunoblot analysis was a complete absence of ADA2 protein in the supernatant from L351Q "ADA2 expressing" CHO cells (**Figure 2A**) and a corresponding intracellular accumulation of L351Q ADA2. Densitometry analysis of band intensity suggests 8.2 +/- 4.5 fold more L351Q ADA2 is retained intracellularly compared to WT ADA2, n = 3, *p* < 0.0001. Although predominantly intracellular, the quantity of L351Q ADA2 protein (supernatant + lysate), unlike G47R ADA2, was similar to WT ADA2. Noteworthy is the presence of L351Q ADA2 dimers (in addition to monomers) that were not observed in comparable abundance for WT (or G47R or W362G) ADA2 protein (**Figures 2A, B**).

To confirm that introduced variants were damaging to ADA2 activity, a colourimetric adenosine deaminase (ADA) activity assay (3, 15) was employed to measure ADA2 catalytic activity of the supernatants (extracellular ADA2; from approximately 2x10^4^ cell equivalents) and lysates (intracellular ADA2; from approximately 2x10^4^ cell equivalents) from the WT and ADA2 variant expressing CHO cells. Results shown in **Figure 2C** are mean total (intracellular and extracellular) ADA2 activity calculated from the sum of activity measured in supernatants and the lysates. As expected, WT ADA2 expressing cells had the highest total (supernatant + lysate) ADA2 activity (16.9 +/- 0.5 U/L; n = 3). Based on extracellular ADA2, measured WT ADA2 catalytic activity values from the CHO cells were comparable to levels detected in human plasma from healthy adults (20). In cells expressing ADA2 containing a W362G substitution in the dimerization domain, total ADA2 activity was significantly reduced (36% of total WT ADA2 catalytic activity; n = 3, *p* < 0.0001) but was not abolished (6.0 +/- 0.3 U/L; n = 3) (**Figure 2C**), and ADA2 activity in supernatants versus lysates was comparable (data not shown). In contrast, introduction of pathogenic variants G47R in the dimerization domain and L351Q in the catalytic domain severely compromised total intracellular and extracellular (supernatant + lysate) ADA2 activity (1.7 +/- 1.2 U/L; n = 3 for G47R and 2.2 +/- 0.4 U/L; n = 3 for L351Q) (**Figures 2C, D** normalized to ADA protein). These results are consistent with units of serum ADA2 activity that we reported in DADA2 patients with these and other pathogenic variants (3).

### N-linked glycosylation enhances stability and secretion of WT, but not L351Q, ADA2

The intracellular retention and accumulation of L351Q ADA2 was consistent with observations of variant-specific differences in ADA2 protein secretion/retention by transfected HEK293 cells as reported by Navon-Elkan et al. (1); in that report, secretion of G47V ADA2 and R169Q ADA2 were substantially compromised compared to WT ADA2 and ADA2 containing variants G47R, P251L and W264S. Although it has been speculated that ADA2 catalytic activity and secretion are dependent, in full or in part, on ADA2 homodimerization (16), commercial and investigator-driven (1, 12, 17) secretion of catalytically active, monomeric recombinant ADA2 by mammalian expression systems (CHO, HEK293, U937) strongly suggests a role for other factors, such as N-linked glycosylation, in regulating ADA2 secretion. There are three predicted N-linked glycosylation sites on ADA2 based on electron density in the crystal structure data (N153, N185, and N378) (16), and an additional putative site (N174) based on in silico analysis of conserved NXS/T consensus sequences (17) (**Figure 1**).

**Figure 3:**
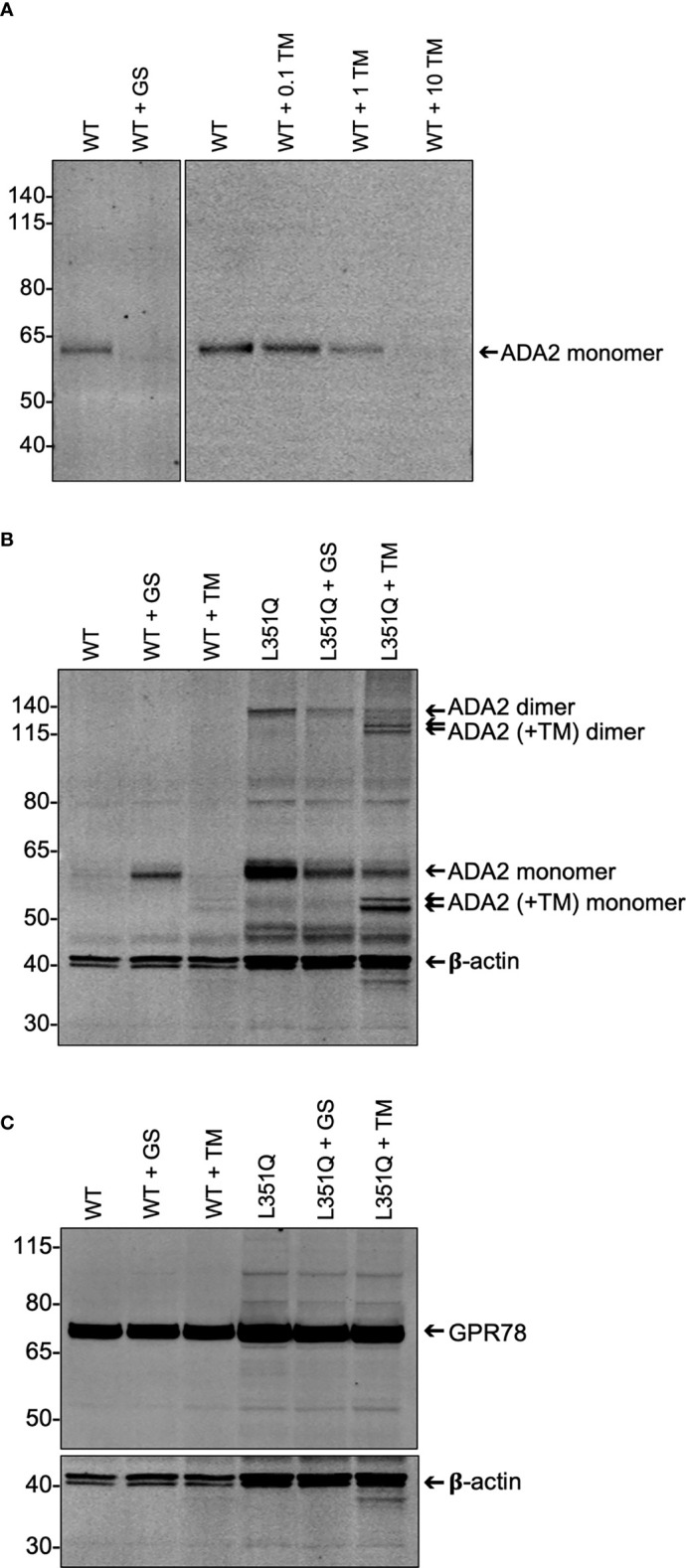
Impact of experimentally-induced inhibition of protein processing on wildtype and L351Q ADA2 expression from Flp-IN CHO cells. **(A)** Representative (n = 3) anti-ADA2 immunoblot of supernatant from wild-type (WT) ADA2 expressing Flp-IN CHO cells treated without (WT), with Golgi transport inhibitor GolgiStop (WT + GS; 5.6 µL/mL) and with N-linked glycosylation inhibitor tunicamycin (WT + TM; 0 – 10 µg/mL). Arrow shows ADA2 monomers (~60 kDa). **(B)** Representative (n = 3) anti-ADA2 immunoblots of lysate (3 x 10^5^ cell equivalents) from Flp-IN CHO cells transfected with expression vectors for WT or L351Q ADA2 and treated with no inhibitor (WT, L351Q), GolgiStop (WT + GS, L351Q + GS; 5.6 µL/mL GolgiStop), or tunicamycin (WT + TM, L351Q + TM; 10 µg/mL tunicamycin). Samples were probed simultaneously for a loading control, beta-actin (β-actin). Arrows show ADA2 monomers and dimers in the absence and presence (+TM) of tunicamycin. **(C)** Representative (n = 3) anti-GPR78 immunoblots of lysate (3 x 10^5^ cell equivalents) from Flp-IN CHO cell samples shown in **(B)**. For **(A-C)**, ladder (left) marks relative molecular weights in kilodaltons (kDa).

We hypothesized that the L351Q variant might impact N-linked glycosylation at the closest Asn (N) residue on the 3-D protein structure (N378; **Figure 1B**), as has been predicted by *in silico* analysis (21). If the L351Q variant disrupts N-linked glycosylation, thereby compromising secretion of ADA2, we reasoned that tunicamycin treatment of CHO cells expressing WT ADA2 would also impede secretion of ADA2 (22). As a control, WT ADA2 expressing cells were treated with GolgiStop (23), which resulted in an expected absence of ADA2 in the cell supernatant (**Figure 3A**) and a corresponding increase in intracellular ADA2 in the lysate of GolgiStop treated cells (**Figure 3B**). Consistent with our hypothesis, treatment of WT ADA2 expressing cells with increasing concentrations of tunicamycin (0.1 – 10 µg/mL for 6 hr) resulted in a dose dependent reduction of ADA2 protein in the cell supernatant to a level that was undetectable (by immunoblotting) following treatment with the highest tested concentration (10 µg/mL) of tunicamycin (**Figure 3A**). In corresponding cell lysates, two (faint) bands below the anticipated molecular weight of the ADA2 monomer were detected (**Figure 3B**). The reduced molecular weight of ADA2 in the presence of tunicamycin is consistent with the resolution of unglycosylated protein. Even so, the absence of N-linked glycans appears to have an overall destabilizing effect on WT ADA2 protein.

This banding pattern of lower molecular mass proteins in tunicamycin-treated cells is more clearly observable in the lysate of L351Q ADA2 expressing CHO cells for both monomers and dimers of ADA2, likely due to overall higher intracellular abundance of L351Q ADA2 protein. While the use of tunicamycin does not allow us to discern the extent of N-linked glycosylation at any particular glycosylation site (e.g. N378), it does provide evidence that N-linked glycans are added to the L351Q variant form of ADA2, even if not at N378. Moreover, ADA2 homodimer formation was not enhanced or reduced compared to untreated L351Q "ADA2 expressing" cells in the presence of GolgiStop or tunicamycin (**Figure 3B**), suggesting that, at least for L351Q ADA2, dimer formation is independent of glycosylation. Finally, these data are consistent with previously published indirect evidence that dimerization is not required for ADA2 secretion (1, 17). It follows that any impact N-linked glycosylation has on ADA2 protein stability and/or secretion is not mediated through altered dimerization.

### Intracellular retention of L351Q ADA2 does not impact cell growth or integrity

Next, we questioned if the intracellular accumulation of L351Q ADA2 had a negative impact on CHO cells that could promote a state of cellular (inflammatory) stress. To do this, we compared CHO cells expressing WT and L351Q ADA2 for evidence of differential growth, toxicity and endoplasmic reticulum (ER) stress.

Using trypan blue exclusion and fluorescence (CFSE) tracing of cell division, no significant differences in cell proliferation were observed between WT and L351Q "ADA2 expressing" CHO cells (**Supplementary Figure S2A**). To assess cellular metabolism and toxicity, we used, respectively, standard assays to quantitate (reduction of) MTT and (extracellular) LDH. No differences in cellular metabolism (MTT) or toxicity (LDH) were observed between CHO cells transfected with WT ADA2 compared to L351Q ADA2, or between these cell lines and CHO cells transfected with empty vector pcDNA5/FRT that confers the same antibiotic resistance and culture conditions (data not shown). In addition, we measured total ADA (ADA1 + ADA2) catalytic activity and calculated ADA1-specific catalytic activity (total ADA activity – ADA2-specific activity) in the cell supernatant (**Supplementary Figure S2B**). In our experience, elevated ADA1 activity in the extracellular space is a sensitive indicator of cell death/inflammatory toxicity. As anticipated, total ADA (ADA1 + ADA2) activity was significantly higher in the supernatant of WT ADA2 expressing cells compared to L351Q ADA2 expressing cells (*p* = 0.0258). Similar to results obtained from the LDH and MTT assays, no differences in ADA1 activity were observed between WT ADA2 and L351Q ADA2 expressing CHO cells (**Supplementary Figure S2B**, left and right panels). Taken together, we find no evidence (derived from MTT assay, LDH quantification, and extracellular ADA1 enzyme activity) to suggest that intracellular accumulation of L351Q ADA2 confers a change in cell metabolism, toxicity and/or membrane leakage.

Finally, we asked if intracellular accumulation of ADA2 was associated with ER stress, as indicated by the differential abundance of glucose-regulated protein 78 (GPR78, also known as binding immunoglobulin protein, BiP). GPR78/BiP is a constitutively expressed, predominantly ER-localized protein of the heat shock protein-70 family (encoded by *Hspa5*) (24). A key function of GPR78/BiP is as a molecular chaperone that aids *de novo* protein folding and oligomerization yet also facilitates elimination of misfolded proteins as part of the unfolded protein (UPR) response (25–27). Although tunicamycin treatment is frequently correlated with elevated GPR78 abundance, GPR78 in WT and L351Q ADA2 (**Figure 3C**) expressing CHO cells was not influenced by tunicamycin treatment. Likewise, and perhaps also unexpected, GPR78 abundance was similar between WT ADA2 and L351Q ADA2 expressing CHO cells despite substantive intracellular retention of L351Q ADA2 compared to WT ADA2, even in the presence of tunicamycin (**Figure 3C**). These findings may suggest that the Flp-IN CHO cell line and/or GPR78 in this cell line are not an appropriate choice for evaluating an ER stress response, or provide supporting evidence that ADA2 protein folding and turnover is in equilibrium despite retention induced by tunicamycin or the introduction of a L351Q variant.

### Constitutive expression of Type I Interferon responsive genes in CHO cells with L351Q ADA2

A growing number of reports show elevated Type I Interferon (IFN) signaling in DADA2 patients (28–31) and spontaneously upregulated Type I IFN-β response in ADA2 siRNA knock down U937 and HUVEC cell lines (32). We therefore evaluated the expression of the interferon induced protein with tetratricopeptide repeats 3 (*IFIT3*) gene and interferon regulatory (transcription) factor 7 (*IRF7*) gene in WT and variant ADA2 expressing CHO cell lines.

**Figure 4:**
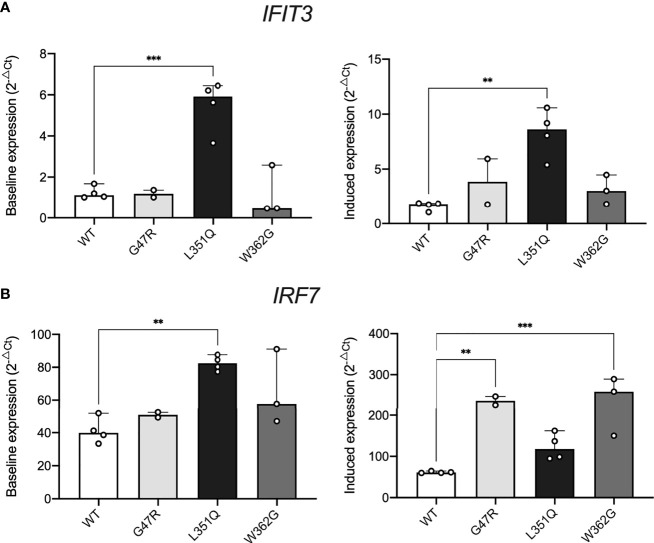
Impact of intracellular L351Q ADA2 on cell stress. Expression of **(A)** *IFIT3* and **(B)** *IRF7* in Flp-IN CHO cells transfected with expression vectors for wild-type (WT, white) and G47R (light grey), L351Q (black), and W362G (dark grey) variant ADA2 cultured with no stimulus (left panels, y-axis; Baseline expression (2^-ΔCt^)) or recombinant human Type I IFNalpha 2b (10 ng/mL, 24 hr) (right panels, y-axis; Induced expression (2^-ΔCt^)). Gene expression was calculated using the delta Ct method (2^-ΔCt^), with eukaryotic *HPRT1* as a housekeeping control; bars show mean + SD (n = 2-4). Statistics show significant results of Dunnett’s multiple comparisons test to compare group means to WT (WT = control). **p ≤ 0.01, ***p ≤ 0.001.

As reported previously (33), we confirmed that Type I IFNalpha 2b (10 ng/mL, 24 hrs) can upregulate *IFIT3* (2.0 +/- 1.2 fold change, *p* = 0.0522) and *IRF7* mRNA (2.1 +/- 1.0 fold change, *p* = 0.0410) in untransfected CHO cells (n = 4). No significant differences in baseline expression of *IFIT3* and *IRF7* (i.e., in the absence of stimulation) were observed between untransfected CHO cells, WT ADA2, G47R ADA2, and W362G ADA2 expressing CHO cells (**Figure 4**). In contrast, baseline expression of both *IFIT3* and *IRF7* were significantly elevated in CHO cells expressing L351Q ADA2 (n = 4, *IFIT3*; 4.6 +/- 1.3 fold higher expression compared to WT ADA2 expressing cells, *p* = 0.0004, *IRF7*; 2.1 +/- 0.4 fold higher expression compared to WT ADA2 expressing cells, *p* = 0.0025).

Stimulation with Type I IFNalpha 2b further upregulated both *IFIT3* and *IRF7* in CHO cells expressing L351Q ADA2 compared to (stimulated) WT ADA2 expressing cells (n = 4, *IFIT3*; 5.1 +/- 0.6 fold higher expression, *p* = 0.0011 and *IRF7*; 2.0 +/- 0.5 fold higher expression, *p* = 0.1329). Although not differentially expressed in resting cells, Type I IFNalpha 2b also induced a significant upregulation of both *IFIT3* and *IRF7* in CHO cells expressing G47R and W362G ADA2 compared to Type I IFNalpha 2b treated WT ADA2 expressing cells (**Figure 4**). These data concur with previous reports of elevated expression of Type I IFN responsive genes in primary human cells and human cell lines devoid of ADA2 catalytic activity. Our results also demonstrate that CHO cells expressing pathogenic catalytic domain variant L351Q ADA2, but not ADA2 dimerization domain variant G47R, is associated with constitutive baseline activation of interferon responsive genes.

## Discussion

In this study, Flp-IN CHO cell lines were generated to stably express WT, G47R or L351Q ADA2 recombinant protein and enable comparative *in vitro* study of the impact of the novel L351Q variant on the ADA2 protein. Our results showing reduced expression of G47R ADA2 (compared to WT ADA2), particularly with inhibition of N-linked glycosylation, are consistent with previous reports in primary cells (2) and the HEK293T mammalian expression system (1). In contrast, our data demonstrates that L351Q ADA2, unlike WT or G47R ADA2, retains the ability to form dimers and is highly stable with and without the incorporation of N-linked glycans. In addition to abrogating catalytic activity, the L351Q variant confers an enzyme-independent, damaging effect on ADA2 by impairing secretion of the protein. While accumulation of intracellular L351Q ADA2 does not alter cellular growth or induce cellular stress in the CHO cells, it was associated with constitutive expression of Type I Interferon stimulated genes.

We considered potential mechanisms, namely the impairment of N-linked glycosylation, by which the L351Q variant might block secretion of human ADA2. The mammalian CHO cells are a useful model for studying protein glycosylation in cell culture, as they have appropriate protein processing machinery to allow for glycosylation patterns similar to those in humans (34). However, differences may exist in human cells. Although L351 is not located in a consensus sequence predicted to be required for N-linked glycosylation, it is in close proximity to N378 (**Figure 1B**) and analysis of a patient with clinical features not unlike those observed in the patients carrying the L351Q variant, including early-onset severe neurological (intracranial hemorrhage) and hematological (anemia) manifestations, revealed a mutation in an amino acid (T129) found in an N-linked glycosylation consensus sequence (NXS/T) (17). Variants that impact one of the N-linked glycosylation sites, or residues in their consensus sequences, have been shown to result in ER retention and defective tracking to the Golgi apparatus (17). Consistent with these observations, we demonstrate herein that total block of N-linked glycosylation impairs secretion of WT ADA2. This decrease in extracellular WT ADA2 was not accompanied by an intracellular accumulation of protein, which may also indicate that loss of N-linked glycosylation, at least for WT and G47R ADA2, leads to preferential targeting of ADA2 for degradation. Our data is inconclusive with regards to whether blocking N-linked glycosylation directly impairs ADA2 enzyme activity, as there was insufficient ADA2 protein in the lysates of tunicamycin-treated cells expressing WT ADA2 to evaluate this. We see clearly, however, that blocking N-linked glycosylation does not impair ADA2 homodimer formation, at least not of L351Q ADA2. We also observed dimeric L351Q ADA2 protein in cells treated with GolgiStop, which blocks protein transport primarily in the trans-Golgi, proximal to the plasma membrane (23). Taken together, these results suggest that the L351Q variant does not impair homodimer formation and is independent of N-linked glycosylation occurring prior to, or early upon entry to, the Golgi apparatus. Thus, impaired incorporation of N-glycans and/or dimerization does not explain impaired secretion or loss of catalytic activity of L351Q ADA2. We reason that this conclusion may also be drawn for WT ADA2, although we do not observe WT ADA2 dimers by immunoblotting.

An accumulation of intracellular protein resulting in a cellular stress response and exacerbation of inflammation is a mechanism that is well described in several inflammatory diseases (35). Although we did not observe classical signs of cellular stress (impaired growth and upregulation of cellular stress) in CHO cells harbouring intracellular L351Q ADA2, our findings show constitutive expression of Type I IFN stimulated genes; (ISGs) this constitutive expression of ISGs was not observed in CHO cells expressing WT, G47R or W362G ADA2. Whether the observed upregulation of ISGs is triggered by the accumulation of intracellular L351Q ADA2 or the disproportionate transport and metabolism of adenosine intracellularly (due to the absence of extracellular, enzymatically active ADA2) that subsequently triggers an IFN-β response (32), remains to be evaluated.

At the onset of this study, we speculated that, given the severe, early-onset hematologic effects observed in individuals with L351Q ADA2, this missense variant would have a detrimental impact on ADA2 function. This notion – that hematologic phenotypes are associated with variants that have a more substantive negative impact on ADA2 function – is aligned with those expressed by Lee et al. (12). While their work has focused solely on extracellular adenosine deaminase (in)activity, we provide new evidence that the L351Q missense variant has detrimental, enzyme-independent effects in the CHO cell model. Our data suggests that the damaging effects of the L351Q variant are due to the intracellular retention of L351Q ADA2 that, in addition to eradicating extracellular ADA2 enzyme activity, results in the constitutive expression of ISGs. It follows that other putative enzyme-independent extracellular function(s) of ADA2 (e.g. growth factor activity) would also be abolished.

*In silico* and functional analyses aimed at elucidating normal molecular functions of ADA2 and the impact of ADA2 variants on function and clinical phenotypes have revealed unexpected differences in the secretion/retention and dimerization of ADA2 (12, 21, 36). For example, particular variants in *ADA2* that disrupt N-linked glycosylation or dimerization, or reduce protein abundance of variant ADA2 (relative to WT) in some expression systems, have been reported (1, 2, 16, 17). Despite these findings, DADA2 clinical phenotypes are often studied with the view that clinical features are due to loss of catalytic functionality and the unrestricted regulation of adenosine and adenosine-mediated immune functions, as seen in ADA-SCID (37). Our findings provide additional evidence that variants in *ADA2* have differential effects on dimerization, stability, and secretion of ADA2 that are not necessarily related to catalytic activity or anticipated based on the location in the protein or the nature (e.g. missense) of the variant. Specifically, we demonstrate that a missense variant in *ADA2* can severely impact protein secretion independent of effects on dimerization, glycosylation and/or protein stability in the CHO cell model. Until we understand whether the clinical manifestations of DADA2 are mediated by pro-inflammatory and potentially toxic effects of adenosine, loss of direct action of ADA2 on cell growth, or both, it is important to consider variant-mediated impacts on ADA2 beyond impaired catalytic activity.

Consistent with other reports, our study shows that dimerization domain-localized variant G47R, but not W362G, impairs the stability and secretion of ADA2. Our study also demonstrates that the L351Q variant has no apparent impact on ADA2 homodimer formation and L351Q ADA2 protein is stable in the absence of N-linked glycosylation. Yet, this missense L351Q variant significantly abrogates ADA2 catalytic activity and impedes ADA2 secretion. The intracellular accumulation of L351Q ADA2 does not appear to induce a general state of cellular stress, but does induce the constitutive expression of Type I Interferon stimulated genes. The impaired secretion of L351Q ADA2, which would simultaneously eliminate enzymatic and non-enzymatic functions of extracellular ADA2, may be an important factor leading to the severe phenotype observed in patients with this variant and further emphasizing the importance of assessing impacts beyond catalytic activity when evaluating genotype-phenotype relationships in DADA2.

## Data availability statement

The raw data supporting the conclusions of this article will be made available by the authors, without undue reservation.

## Author contributions

SB: data generation and interpretation, creation of figures and tables and manuscript preparation. MS: data generation and interpretation, and manuscript preparation. DC and PD: clinical oversight of patients and manuscript preparation. KB: study design, overview of data generation and interpretation, and manuscript preparation. All authors read and approved the final manuscript.

## Funding

This study was funded by Canadian Institutes of Health Research (CIHR) team grant [TR2-119188 to DA]. The content is solely the responsibility of the authors and does not necessarily represent the official views of the Canadian Institutes for Health Research. SB was funded by a University of British Columbia (UBC) Faculty of Medicine Summer Student Research Award, Centre for Blood Research (CBR) summer student program fellowship, the UBC affiliated Andrew Nord Fellowship in Rheumatology, and is currently funded by a CIHR Canada Graduate Scholarships Doctoral (CGS D) Award. MS received funding from the VGR MoRE2020 fellowship co-funded by the European Union’s Horizon 2020 research and innovation programme under the Marie Skłodowska-Curie grant agreement No 754412. KB is supported by a Michael Smith Foundation for Health Research Scholar Award. DC is supported by The Arthritis Society (TAS) Canada through the Ross Petty Arthritis Society Chair.

## Acknowledgments

The authors thank the UBC Centre for Molecular Medicine and Therapeutics DNA Sequencing Core Facility for their plasmid sequencing services. We also thank Kristen Gibson, Stephanie Hughes, Dilpreet Bharaj, and Dr Ross Petty for their outstanding technical support.

## Conflict of interest

The authors declare that the research was conducted in the absence of any commercial or financial relationships that could be construed as a potential conflict of interest.

## Publisher’s note

All claims expressed in this article are solely those of the authors and do not necessarily represent those of their affiliated organizations, or those of the publisher, the editors and the reviewers. Any product that may be evaluated in this article, or claim that may be made by its manufacturer, is not guaranteed or endorsed by the publisher.
