# Supplementary material for: Pathogenic variant c.1052T>A (p.Leu351Gln) in adenosine deaminase 2 impairs secretion and elevates type I IFN responsive gene expression
Source: Front Immunol. 2022 Sep 30;13:995191. doi: 10.3389/fimmu.2022.995191 (PMC9562767; doi:10.3389/fimmu.2022.995191)
Supplement: Supplementary file 1 [file DataSheet_1.docx]

# Supplementary Tables and Figures

**Supplementary Table 1.** *Non-overlapping mutagenic primers designed for site directed mutagenesis using the NEBase Changer web tool.*

| **Name** | **Sequence^*^** | **Consequence** |
| --- | --- | --- |
| ADA2_G139C_Fwd | GATGCGGCTGcGGGGGCGGCT | p.Gly47Arg |
| ADA2_G139C_Rev | ATCTTTTCTTTCAACAACAGATGCGCCCGTG | p.Gly47Arg |
| ADA2_T1052A_Fwd | GGCGTTAAGCaGCCTTACTTC | p.Leu351Gln |
| ADA2_T1052A_Rev | ATCCTTGGCGGGGATCAT | p.Leu351Gln |
| ADA2_T1084G_Fwd | AGAAACAGACgGGCAGGGTAC | p.Trp362Gly |
| ADA2_T1084G_Rev | CCGGCGTGGAAGAAGTAA | p.Trp362Gly |

*Lower case letter denotes variant.

**Supplementary Figure 1.** ADA2 *protein expression and ADA2 and total ADA catalytic activity in untransfected Flp-IN CHO cells*. ***A***) Simultaneous anti-ADA2 and anti-beta actin (β-actin; loading control) immunoblot of lysate (3x10^5^ cell equivalents) from Flp-IN CHO cells untransfected (Untr. lys; right lane) and with the expression vector for L351Q ADA2 (L351Q lys; left lane) (3 technical replicates). Ladder (right) marks relative molecular weights in kilodaltons (kDa) and ADA2 dimers (>120 kDa), ADA2 monomers (~60 kDa), and putative ADA-like proteins (<50 kDa) are indicated by arrows (left). ***B***) ADA2 catalytic activity (white bars; measured in the presence of EHNA) and total adenosine deaminase (ADA) catalytic activity (grey bars; measured in the absence of EHNA) in supernatant containing cellular lysates from untransfected (Untr), and wild-type (WT) and variant (L351Q) ADA2 expressing, Flp-IN CHO cells (3 technical replicates). To generate supernatant containing cellular lysates for measuring intra and extracellular ADA activity simultaneously, Triton X-100 and protease inhibitor cocktail was added directly to adherent cells and media (final concentration 1% Triton X-100) following 6 hr growth at 2x10^6^ cells/mL. Following incubation (20 min), supernatant with lysed cells was collected and cleared by centrifugation at 4°C (16,000x*g* for 10 min), and 10 uL (approximately 2x10^4^ cell equivalents) was used for the activity assay. Dotted line shows upper limit of quantification (ULOQ) of the activity assay (30 U/L). Bars show mean + SD and statistics show results of Dunnett’s multiple comparisons test to compare group means to Untr (Untr = control) for ADA2 (+ EHNA) enzyme activity only. ns = nonsignificant, ** *p* ≤ 0.01.

**
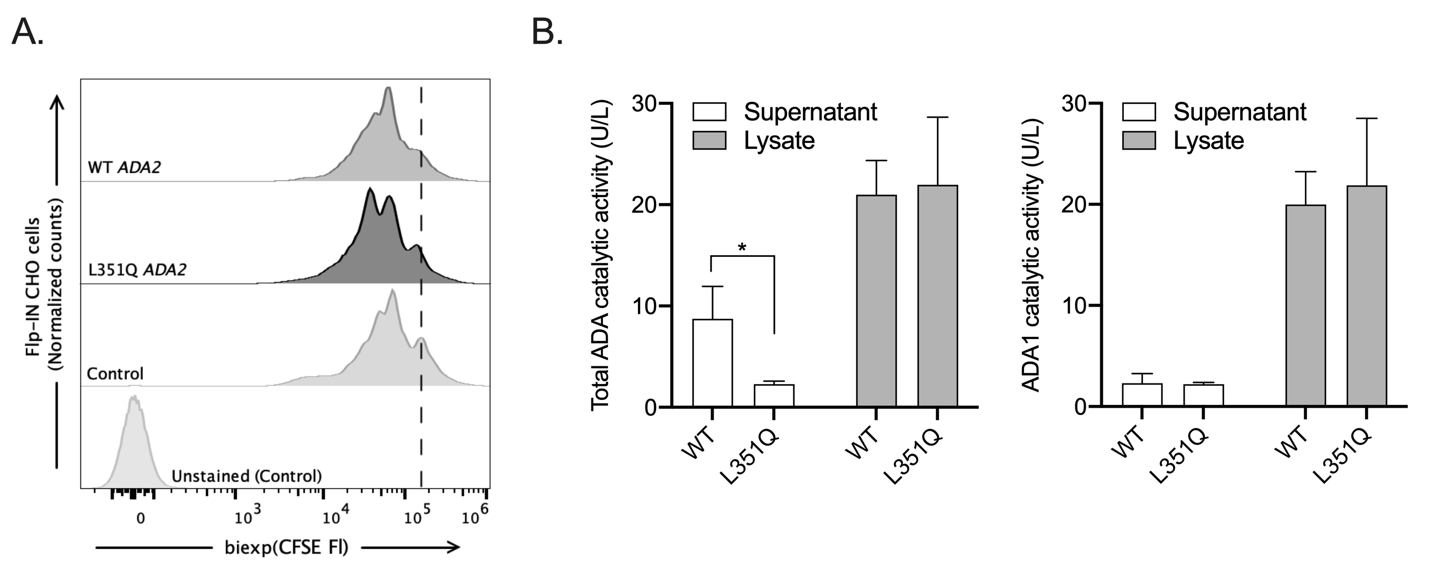
**

**Supplementary Figure 2.** *Comparison of relative proliferation rates of transfected Flp-IN CHO cells.* ***A)*** Fluorescence of carboxyfluorescein succinimidyl ester (CFSE) labelled Flp-IN CHO cells (left panel) transfected with the pcDNA5/FRT vector for expressing WT or L351Q *ADA2* (top two rows; representative of n = 2), or control cells transfected with the empty pcDNA5/FRT vector (second row from bottom; n = 1) after 48 hr of growth. Unstained, control cells are shown in the bottom row (n = 1). Cells were initially stained using the Vybrant CFSE cell tracer kit (Thermo Fisher Scientific, MA, USA) at 1x10^6^ cells/mL (10 μM CFSE), seeded in a 12-well plate (1.5x10^5^ cells per well) and incubated 48 hr prior to analysis on an Attune NxT Flow Cytometer (Thermo Fisher, MA, USA). A minimum of 2x10^4^ events were collected (x-axis: biexponential (biexp) transformation of CFSE fluorescence (CFSE Fl)), and data were analyzed using FlowJo^TM^ software (v.10.6.2; BD Biociences, NJ, USA). Flow cytometry data are supported by Trypan Blue exclusion counts. ***B)*** Total adenosine deaminase (total ADA) catalytic activity (y-axis; total ADA catalytic activity (U/L), n = 3) measured in the absence of EHNA (left panel) and calculated adenosine deaminase 1 (ADA1; total ADA – ADA2) catalytic activity (y-axis; ADA1 catalytic activity (U/L), n = 3) (right panel), in supernatant (white) and lysate (light grey) from Flp-IN CHO cells transfected with expression vectors for WT and variant (L351Q) ADA2*.* Bars show mean + SD and statistics show significant results of unpaired t tests to compare WT and L351Q means within supernatant and lysate, respectively. **p* ≤ 0.05.
